# Prognostic Value of C4d Immunolabelling in Adult Patients With IgA Vasculitis

**DOI:** 10.3389/fmed.2021.735775

**Published:** 2021-11-29

**Authors:** Anais Romero, Fanny Drieux, Arnaud François, Alexandra Dervaux, Xiao Li Xu, Dimitri Titeca-Beauport, Dominique Bertrand, Dominique Guerrot

**Affiliations:** ^1^Department of Nephrology, Hemodialysis, Kidney Transplantation, Rouen University Hospital, Rouen, France; ^2^Pathology Department, Centre Henri Becquerel, Rouen, France; ^3^Pathology Department, Amiens University Hospital, Amiens, France; ^4^Department of Nephrology, Hemodialysis, Renal Transplant, Amiens University Hospital, Amiens, France; ^5^UNIROUEN, INSERM U1096, Rouen, France

**Keywords:** immunoglobulin A (IgA), vasculitis, glomerulonephritis, Henoch-Schönlein purpura, immunohistochemistry, proteinuria (MeSH: D011507), complement

## Abstract

**Background and Objectives:** Glomerular C4d deposits are associated the severity and outcomes of IgA nephropathy. Whether this holds true in immunoglobulin A vasculitis (IgAV) is not known. The main objective of the study was to analyze the prognostic value of glomerular C4d immunolabelling on kidney impairment in adults with IgAV.

**Design, Setting, Participants, Measurements:** This retrospective cohort study included 120 adults with IgAV and a kidney biopsy performed between 1995 and 2018 in two French university hospital centers. All paraffin-embedded biopsies were reassessed according to Oxford classification. Immunofluorescence for C4d was performed in all cases. For analysis, patients were grouped according to positivity for C4d in the glomerular area. The main outcome was a composite endpoint of 50% increase in 24 h-proteinuria, or eGFR decrease by 50%, or kidney replacement therapy.

**Results:** The median follow-up was 28.3 months. Twenty-three patients met the composite endpoint, 12 for kidney replacement therapy, 6 for an eGFR decrease >50% and 5 for a >50% increase in proteinuria. At time of biopsy, the median proteinuria was 1.9 g/24 h and the median eGFR 73.5 mL/min/1.73 m^2^. Among the 102 patients evaluable for C4d, 24 were positive on >30% glomeruli, mainly with a parieto-mesangial pattern. In this group, the initial proteinuria was more frequently nephrotic than in the C4d– group (60% vs. 33%, *P* = 0.039). Mesangial hypercellularity was more frequent in the C4d+ group (42% vs. 13%; *P* = 0.006) whereas macroscopic hematuria was more frequent in the C4d– group (18% vs. 0%; *P* = 0.03). After a median follow-up of 28 months, kidney survival did not differ according to C4d status.

**Conclusion:** In a population of adult IgAV patients, glomerular positivity for C4d was associated with the severity of the kidney disease at presentation, but not with subsequent renal function deterioration.

## Introduction

Immunoglobulin A vasculitis (IgAV), formerly called Henoch-Schönlein purpura, is an immune complex vasculitis predominantly affecting small vessels with dominant IgA deposits. It can affect the skin, gut, joints and kidney to varying degrees. IgA vasculitis is considered to be a systemic form of IgA nephropathy and it has been suggested that the two conditions are different manifestations of a single disease process (1). A kidney involvement is more frequent and severe in adults than in children. As outlined by Audemard-Verger et al. (2), a kidney involvement occurs in 45–85% of cases, but IgA vasculitis is responsible for only 0.6–2% of adult nephropathies.

In addition to IgA deposits, glomerular deposition of complement factors including C3, mannan-binding lectin, L-ficolin, mannan-associated serine protease, and C4d has been observed in most patients with IgA vasculitis. These findings, together with the absence of C1q deposits, support the hypothesis of prevalent activation of the lectin pathway. The presence of complement deposits has been shown to be associated with a higher degree of proteinuria and hematuria as well as with more severe histological lesions (3).

C4d is a biological degradation product of the C4 fraction of complement after activation of the conventional or the lectin pathway, with no known biological function or receptor. The presence of C4d deposits has been reported in IgA nephropathy in association with mannose binding lectin deposits, indicating, in the absence of C1q, an activation of the lectin pathway (4, 5). Several publications have shown that the presence of mesangial glomerular deposits of C4d in IgA nephropathy is associated with a more severe form of the disease and an adverse prognosis (6–8).

The involvement of the lectin pathway has also been shown in IgA vasculitis, associated with an increase in serum C4d (5), but the prognostic interest of C4d immunostaining in the kidney has not yet been studied.

The objectives of the study were to analyze the presence and prognostic value of C4d deposits in the kidney in adult patients with IgA vasculitis.

## Materials and Methods

This retrospective cohort study was conducted in two French University Hospitals (Rouen and Amiens). In French hospital settings, patients are informed that their data can be used for research purposes if they have no objection. The data used in this study are derived from de-identified files, and thus, this study was exempt from Ethics Committee approval.

We retrospectively identified all persons 18-year old or older who did not object to the use of their data, who underwent a kidney biopsy between January 1995 and January 2018, were found to have glomerular IgA deposition on histopathological examination and had at least one extrarenal sign compatible with IgAV. Patients with liver disease, digestive or articular chronic inflammatory disease, primary IgA nephropathy, lack of dominant IgA mesangial deposits or lack of material were excluded.

### Patients Follow-Up

Clinical and biological data were extracted from the pathology laboratories information system and electronic medical records. Patients' follow-up ran from the date of biopsy to the date of last visit, death or kidney replacement therapy.

Demographic data, hypertension status, extrarenal signs (cutaneous purpura, arthralgia, abdominal pain, digestive hemorrhage), macroscopic or microscopic hematuria, plasma creatinine, glomerular filtration rate estimated by the MDRD formula (eGFR), urinary protein excretion, and treatment with Angiotensin-Converting Enzyme Inhibitors (ACEi) or Angiotensin II Receptor Blockers (ARB) were recorded at kidney biopsy. Follow-up data included plasma creatinine and proteinuria after 1, 2 and 5 years and at last visit, treatment with immunosuppressive, corticosteroid or ACEi/ARB agents, and death or relapse defined by purpura resurgence or proteinuria >0.5 g/24 h.

The main outcome was a composite endpoint of 50% increase in 24 h-proteinuria, or eGFR decrease by 50%, or kidney replacement therapy.

### Histopathology of Kidney Biopsies and Immunostaining

All biopsies were evaluated by an independent pathologist blinded to clinical outcomes. Paraffin-embedded kidney tissues (2-μm sections) were stained with Hemalun Eosine Safran, Masson trichrome, Jones methenamine silver and sometimes periodic acid–Schiff and Congo red. To be analyzed, biopsies had to contain at least five glomeruli. They were classified according to the Oxford criteria (9). Additionally, presence of extra-capillary proliferation, fibrinoid necrosis and moderate to severe arteriosclerosis were evaluated.

C4d immunohistochemical staining was performed on 2-μm sections of renal tissue using rabbit polyclonal anti-human C4d (clone A24-T, DB Biotech, Kosice, Slovakia) according to Ventana protocol. Only biopsies with at least four permeable glomeruli were analyzed. The positivity for C4d staining was defined as more than 30% of non-sclerotic glomeruli with a moderate or strong staining. The staining was global if involving more than 50% of the glomerular tuft.

### Statistical Analysis

The number of cases retrieved determined the study size. Statistics were performed using R software (10). Quantitative variables were expressed as median with their interquartile range (IQR) and compared between C4d+ and C4d– groups with the Mann-Whitney U test; Categorical variables were described as absolute numbers and percentages and compared between C4d+ and C4d– groups using the Fisher's exact test. Kidney survival was analyzed using Kaplan-Meier estimates and comparisons between C4d+ and C4d groups used a log-rank test. Missing data were not replaced.

## Results

**Figure 1:**
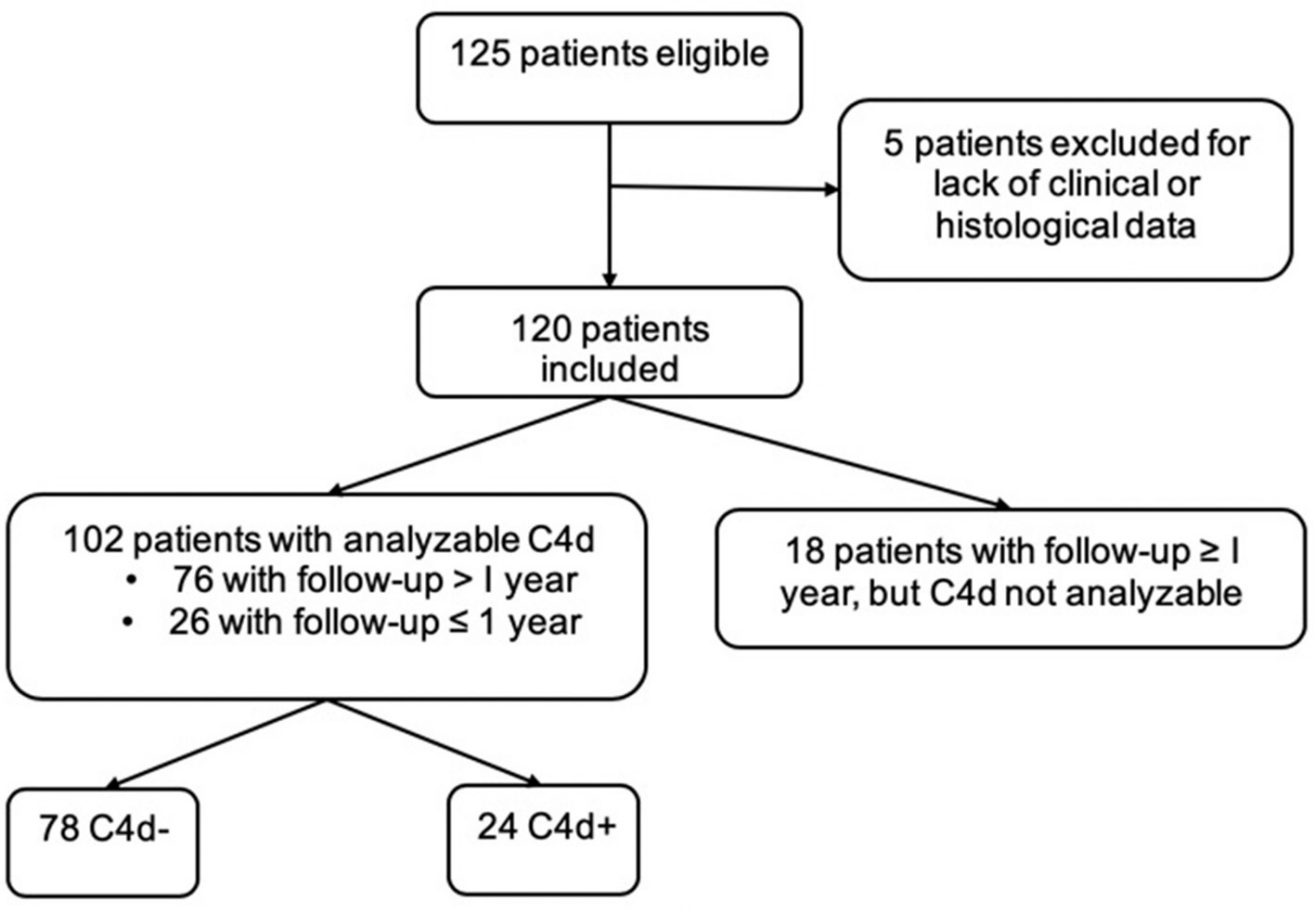
Patient flow-chart.

This retrospective cohort included 120 patients biopsied and diagnosed with IgA vasculitis. A flow diagram describing the patient samples and exclusions is shown in Figure 1.

### Baseline Characteristics and Follow-Up of the Overall Population

**Figure 2:**
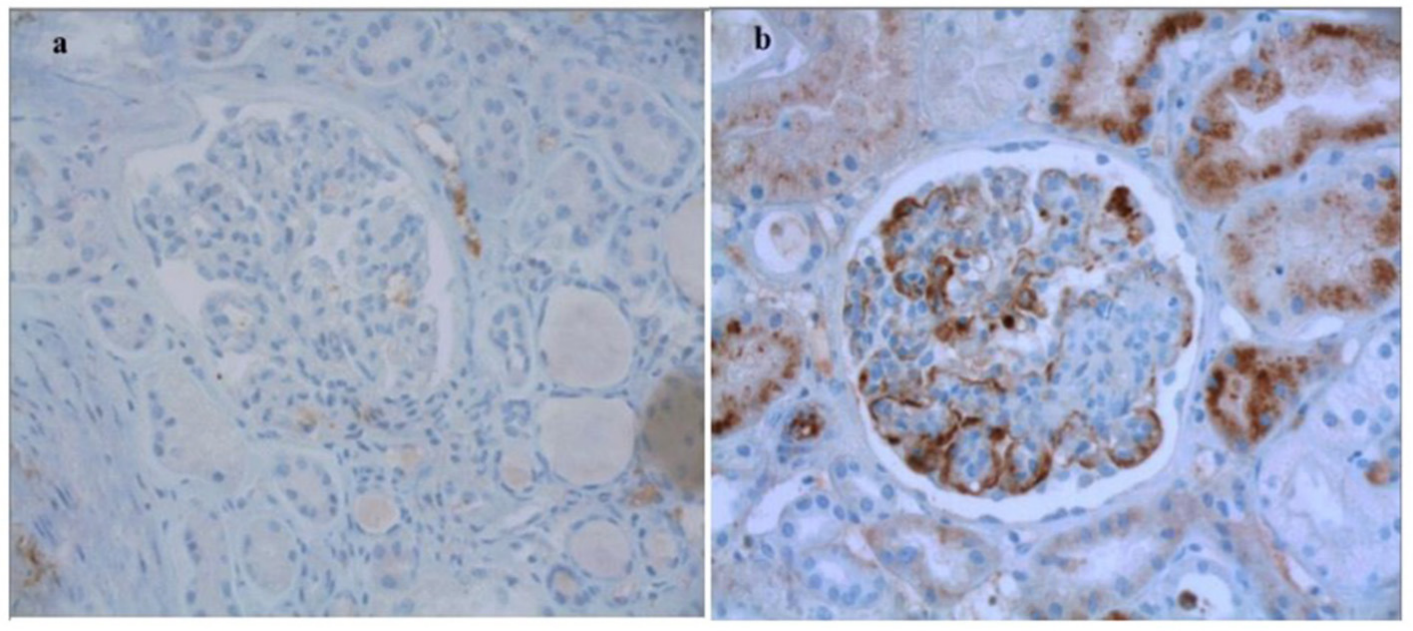
Representative views of C4d immunohistochemistry on kidney biospies of patients with IgAV. **(a)** Absence of staining. **(b)** Positive parietal staining. Original magnification x400.

Table 1 shows the clinical and histopathologic characteristics at the time of diagnosis in the whole group and stratified by the presence/absence of glomerular C4d deposits on the kidney biopsy. Three patients were already on kidney replacement therapy at time of diagnosis, none had undergone a kidney transplantation. The activity of the disease was demonstrated by endocapillary inflammation E1 (73.3%), extra-capillary proliferation (66.7%) and fibrinoid necrosis (54.2%), whereas mesangial proliferation M1 was found in 21% of the patients only. At time of diagnosis, chronic lesions were already frequent, as shown by mesangial sclerosis S1 (57.5%), interstitial fibrosis and tubular atrophy T1-T2 (26.7%) and moderate to severe arteriosclerosis (61%). Immunofluorescence was available for 114 patients; all biopsies showed IgA deposits, mesangial in 50% of the cases and parieto-mesangial in 35%, and C3 deposits were found in 83% biopsies. C1q staining was negative in all patients, IgG and IgM staining were positive in respectively 23% and 41% patients. Deposits of C4d were observed in 24 (24%) patients among the 102 biopsies analyzable. In these patients, C4d staining was mainly parieto-mesangial (67%), mostly of moderate intensity (92%) (Figure 2).

**Table 1:** Clinical and histopathological characteristics at diagnosis, overall and according to the presence or absence of C4d glomerular deposits.

	**All** **(*N* = 102)**	**C4d+** **(*N* = 24)**	**C4d–** **(*N* = 78)**	***P*-value**
Age (years); median (IQ)	53 (32–68)	41 (20–62)	54 (35–69)	0.07
Men; n/N (%)*	78/120 (65%)	11/24 (45.8)	52/78 (66.7)	0.09
Extra-renal signs; n/N (%)				
Cutaneous	114/118 (96.6)	21/22 (95.5)	76/78 (97.4)	0.56
Abdominal pain	50/114 (43.9)	12/22 (54.5)	32/74 (43.2)	0.47
Gastrointestinal hemorrhage	18/112 (16.0)	3/21 (14.3)	11/73 (15.1)	1
Arthralgia	63/113 (55.8)	15/22 (68.2)	42/73 (57.5)	0.46
Hypertension; n/N (%)	56/115 (48.9)	7/21 (33.3)	36/76 (47.4)	0.32
eGFR (mL/min/1.73 m^2^); median (IQR)	73 (37–98)	87 (43–113)	69 (29–92)	0.09
Creatininemia (μmol/L); median (IQR)	88 (70–152)	77 (67–120)	91 (70–200)	0.17
Proteinuria (g/24 h)	1.9 (1–3.97)	3.7 (1.06–7.03)	2.0 (1.05–3.8)	0.16
Proteinuria > 3 g/24 h; n (%)*	40/108 (37)	12/20 (60.0)	23/71 (32.3)	0.04
Hematuria; n/N (%)*				0.0024
Macroscopic	16/120 (13.3)	0	14/78 (18.2)	
Microscopic	96/120 (82.8)	19/22 (86.4)	62/78 (80.5)	
Treatment
ACEi/ARB treatment; n/N (%)	0.79	16/22 (72.7)	47/69 (68.1)	0.79
Corticosteroid oral; n/N (%)	0.35	20/22 (90.9)	56/69 (81.1)	0.35
Corticosteroid IV; n/N (%)	1	11/22 (50.0)	45/91 (49.4)	1
Immunosuppressive; n/N (%)	0.054	7/22 (31.8)	9/70 (12.8)	0.054
Histopathologic Oxford classification; n (%)				
M1*	20 (19.6)	10 (41.6)	10 (12.8)	0.006
S1	60 (58.8)	14 (58.3)	46 (58.9)	1
E1	77 (75.5)	19 (79.2)	58 (74.4)	0.79
T1-2	24 (23.5)	8 (33.0)	16 (20.5)	0.27
C1-2	72 (70.5)	17 (70.8)	55 (70.5)	0.95
Other histopathologic findings; n (%)				
Fibrinoid necrosis	54 (52.9)	16 (66.7)	38 (51.3)	0.24
Moderate/severe arteriosclerosis	60 (58.8)	12 (50.0)	48 (61.5)	0.29
C4d distribution				
Number of glomeruli; median (IQR)	11.5 (8–19)	14.5 (10–19.5)	11.0 (7.25–18.5)	0.11
% of affected glomeruli; median (IQR)	52.5 (25–80)	83.5 (60–100)	37.0 (20–67.8)	<0.001
C4d repartition*				0.002
Parietal; n (%)	18 (17.6)	7 (29.2)	11 (17.7)	
Mesangial; n (%)	21 (20.6)	1 (4.2)	20 (32.2)	
Parieto-mesangial; n (%)	47 (46)	16 (66.7)	31 (50)	

*IQR, interquartile range; ACEi, Angiotensin-converting enzyme inhibitors; ARB, Angiotensin II receptor blockers; IV, intravenous; M, mesangial cellularity; S, segmental sclerosis; E, endocapillary hypercellularity; T, interstitial fibrosis and tubular atrophy; T1 > 25% and T2 > 50%*.

* *Statistically significant difference between C4d+ and C4d– groups*.

**Figure 3:**
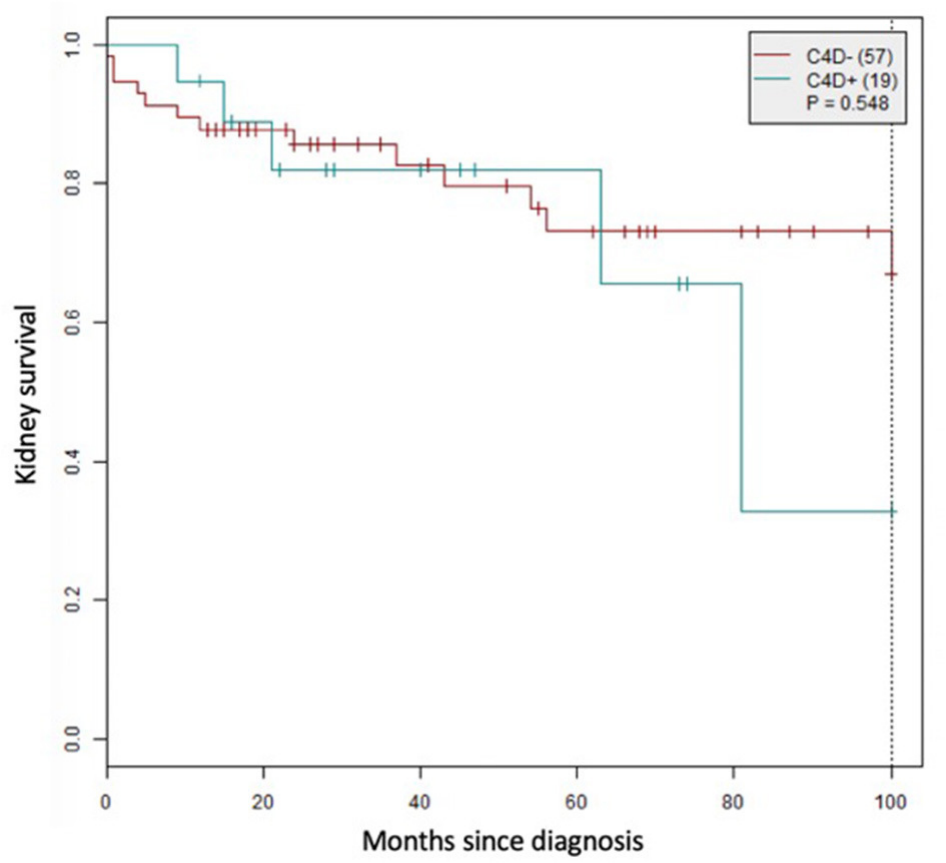
Kidney survival according to glomerular C4d status.

The median follow-up was 28.3 months (IQR 13.8–70). Twenty-three patients (19%) met the composite endpoint, 12 for kidney replacement therapy, 6 for an eGFR decrease >50% and 5 for proteinuria increase >50% (Tables 2, 3; Figure 3).

**Table 2:** Follow-up according to the presence or absence of C4d glomerular deposits.

	**C4d+** **(*N* = 24)**	**C4d–** **(*N* = 78)**	***P*-value**
Follow-up duration (months); median (IQR)	22 (12–65)	29 (10–81)	0.54
Follow-up duration in classes; n (%)			
<1 year	5/24 (20.8)	15/65 (23)	1
[1–2 years[	7/23 (30.4)	9/66 (13.6)	0.11
[2–5 years[	6/23 (26.1)	19/66 (28.8)	1
≥5 years	5/24 (20.8)	23/66 (34.8)	0.3
Composite endpoint met			
Missing	5	20	
n (%)	6 (32)	14 (25)	0.56
Kidney replacement therapy	3	7	0.7
eGFR decrease >50%	1	4	1
Proteinuria increase >50%	2	3	0.59
Time to kidney replacement therapy (months); median (IQR)	15 (12–18)	4 (1–7)	0.13
Relapse			
Missing	1	11	0.51
n (%)	6 (26)	13 (19)	
Death			
Missing	1	20	0.67
n (%)	1 (4)	7 (12)	
5-year survival	14/19 (73.7)	34/57 (59.6)	0.41

*IQR, interquartile range; Relapse = purpura resurgence or proteinuria > 0.5 g/24 h*.

**Table 3:** Follow-up in a sub-group of patients with 2-year available eGFR data according to the presence or absence of C4d glomerular deposits.

	**C4d+** **(*N* = 11)**	**C4d–** **(*N* = 34)**	***P*-value**
Follow-up duration (months); median (IQR)	45 (28–55)	33 (24–89)	0.92
Kidney replacement therapy; n (%)	1 (9)	6 (18)	0.49
Death	0 (0)	5 (15)	0.17
Changes in eGFR (ml/min/1.73 m^2^); median (IQR)			
Baseline	76 (48–120)	56 (23–82)	0.08
1 year	76 (55–92)	68 (38–81)	0.15
2 years	72 (47–81)	68 (35–82)	0.57
Final	74 (57–81)	62 (30–83)	0.26
Changes in proteinuria (g/24 h); median (IQR)			
Baseline	6.8 (3.1–7.6)	2.0(1.0–3.4)	0.03
1 year	0.2 (0.1–1)	0.4 (0–0.7)	0.15
2 years	0.07 (0–0.4)	0.34 (0–0.6)	0.33
Final	0.05 (0–0.4)	0.25 (0.07–0.4)	0.35

*IQR, Interquartile range*.

### Differences Between C4d+ and C4d– Patients

A hundred and two patients were analyzable for C4d. Comparisons of clinical and histopathologic characteristics at the time of diagnosis are displayed in Table 1. Men were more represented and macroscopic hematuria significantly more frequent in the C4d– group. Sixty per cent patients in the C4d+ group had a nephrotic-range proteinuria (>3 g/24 h), twice more than in C4d–, although the median proteinuria did not differ significantly between groups. Treatment by an immunosuppressive therapy was more prevalent in C4d+ patients (31.8%) compared to C4d– patients (12.8%). Mesangial hypercellularity affected 42% of C4d+ patients vs. 13% of C4d– patients, while the other histopathologic findings were similar in the two groups. Immunofluorescence was available for 98 patients; all biopsies showed deposits of IgA, exclusively mesangial in 39% of C4d+ and 53% C4d– cases, parieto-mesangial in respectively 39% and 35% of the C4d+ and C4d– cases. C1q staining was negative in all patients and C3 deposits were found in respectively 91% and 85% of the C4d+ and C4d– biopsies. IgG staining was positive in respectively 41% and 19% of the C4d+ and C4d– patients and IgM in 39% and 40%. C4d immunostaining distribution is summarized in Table 1. The rate of affected glomeruli was significantly higher in the C4d+ group, with a diffuse pattern, whereas deposits were focal in the C4d–. The C4d pattern was significantly different between the two groups, mainly due to lack of exclusive mesangial localization in the C4d+ patients.

Follow-up comparisons are summarized in Table 2. The follow-up length was similar for the two groups, and >1 year for three quarters of the patients. The rate of patients meeting the composite endpoint was not different between groups, as for the number of relapses or deaths. The renal survival did not differ significantly between groups (Figure 3). In the subgroup of patients with available eGFR at 2 years (Table 3), only the baseline values of proteinuria differed between C4d+ and C4d–, changes over time were similar.

## Discussion

In our study, the presence of C4d deposits in the kidney biopsy of adult patients with IgA vasculitis renal impairment was associated with a more severe initial renal involvement: a nephrotic proteinuria >3 g/24 h was reported for 60% patients and mesangial hypercellularity was more frequent, but our results did not show an association between C4d deposits and renal survival. Interestingly, there was a higher proportion of immunosuppressive treatment in the C4d+ group (31.8%) compared to the C4d– group (12.8%), suggesting a more aggressive therapeutical approach in C4d+ patients.

Various clinical, biological and histological factors, as hypertension, high proteinuria and/or plasma creatinine, glomerulosclerosis or interstitial fibrosis, are recognized predictors of adverse kidney outcomes (11, 12). However, progression to kidney failure can occur even in the absence of these poor prognosis factors, underlining the necessity of new prognostic markers.

C4d deposits, generated by the activation of the lectin pathway, appear to be associated with a worse renal prognosis, regardless of the nephropathy studied. Xing et al. (13) were the first to suggest that a positive staining of C4d and mannan-binding lectin might be associated with poor renal outcome. The prognostic interest of C4d immunostaining has been described in IgA nephropathy and identified as an independent risk factor for progression to end-stage renal failure (6, 14) but data on C4d and IgA vasculitis are scarce. In their series of 59 patients, Espinosa et al. (6) included 8 patients with IgA vasculitis, of which 2 were positive for C4d. The frequency of C4d deposition was the same between patients with vasculitis and those with nephropathy. As a possible prognostic factor, C4d could be assessed routinely in kidney biopsies in the same way as C3 or C1q.

In our cohort, C4d positivity was not associated with a more severe extra-renal picture. However, kidney involvement was more severe in the C4d+ group, as shown by worse plasma creatinine and proteinuria, especially nephrotic proteinuria. We did not show a difference in renal survival between the two groups, maybe because of a too short follow-up and a more aggressive treatment in C4d+ patients. In studies on the prognostic value of C4d deposits in IgA nephropathy, the survival curves separate after 5 years of follow-up (6, 7, 15).

We used the Oxford classification to characterize our biopsies: these criteria are prognostic factors for both IgA nephritis and vasculitis, and they are easily reproducible as most often binary. Xu et al. (16) showed the relevance of this classification for predicting long-term outcomes of IgA vasculitis. We found a more frequent mesangial proliferation in the C4d+ group (42% vs. 13% in the C4d– group). Our findings of more frequent nephrotic syndrome in the C4d+ group is in line with the findings of Xu et al. (16), who showed that mesangial proliferation was associated with a worse proteinuria.

Our study has several limitations: the retrospective design is a critical limitation, as all confounding factors might not be identified and distributed equally, which may lead to biased results. Our median follow-up was 28 months, shorter than other studies (11, 12, 17). Given the absence of correlation between initial presentation and long-term renal outcome, with possible spontaneous remission in patients with severe presentation, or possible evolution to end-stage renal disease in patients with mild symptoms (18), we cannot exclude that a longer period of observation may have revealed a discriminant value of C4d deposits.

In conclusion this study, which analyzes the largest cohort of IgAV patients in this setting, demonstrates an association between glomerular positivity for C4d and the severity of the kidney disease at presentation, without showing prognostic value on kidney outcomes after a median follow-up of 28 months.

## Data Availability Statement

The raw data supporting the conclusions of this article will be made available by the authors, without undue reservation.

## Ethics Statement

Ethical review and approval was not required for the study on human participants in accordance with the local legislation and institutional requirements. Written informed consent for participation was not required for this study in accordance with the national legislation and the institutional requirements.

## Author Contributions

AR collected data, performed pathology analysis, analyzed results, performed statistical analyses, wrote the draft, and reviewed the manuscript. FD performed pathology analysis, analyzed results, and reviewed the manuscript. AF and AD performed pathology analysis and reviewed the manuscript. XX and DT-B collected data and reviewed the manuscript. DB analyzed results, performed statistical analyses, and reviewed the manuscript. DG initiated the study, analyzed results, wrote the draft, and reviewed the manuscript. All authors contributed to the article and approved the submitted version.

## Conflict of Interest

The authors declare that the research was conducted in the absence of any commercial or financial relationships that could be construed as a potential conflict of interest.

## Publisher's Note

All claims expressed in this article are solely those of the authors and do not necessarily represent those of their affiliated organizations, or those of the publisher, the editors and the reviewers. Any product that may be evaluated in this article, or claim that may be made by its manufacturer, is not guaranteed or endorsed by the publisher.
